# Ascorbate supplementation inhibits growth and metastasis of B16FO melanoma and 4T1 breast cancer cells in vitamin C-deficient mice

**DOI:** 10.3892/ijo.2012.1712

**Published:** 2012-11-21

**Authors:** JOHN CHA, M. WAHEED ROOMI, VADIM IVANOV, TATIANA KALINOVSKY, ALEKSANDRA NIEDZWIECKI, MATTHIAS RATH

**Affiliations:** Dr Rath Research Institute, 1260 Memorex Drive, Santa Clara, CA 95050, USA

**Keywords:** ascorbate, gulonolactone oxidase knockout mice, metastasis, tumor growth, melanoma B16F0, breast cancer 4T1, collagen I and IV, matrix metalloproteinase-9, apoptosis, interleukin-6

## Abstract

Degradation of the extracellular matrix (ECM) plays a critical role in the formation of tumors and metastasis and has been found to correlate with the aggressiveness of tumor growth and invasiveness of cancer. Ascorbic acid, which is known to be essential for the structural integrity of the intercellular matrix, is not produced by humans and must be obtained from the diet. Cancer patients have been shown to have very low reserves of ascorbic acid. Our main objective was to determine the effect of ascorbate supplementation on metastasis, tumor growth and tumor immunohistochemistry in mice unable to synthesize ascorbic acid [gulonolactone oxidase (gulo) knockout (KO)] when challenged with B16FO melanoma or 4T1 breast cancer cells. Gulo KO female mice 36-38 weeks of age were deprived of or maintained on ascorbate in food and water for 4 weeks prior to and 2 weeks post intraperitoneal (IP) injection of 5×10^5^ B16FO murine melanoma cells or to injection of 5×10^5^ 4T1 breast cancer cells into the mammary pad of mice. Ascorbate-supplemented gulo KO mice injected with B16FO melanoma cells demonstrated significant reduction (by 71%, p=0.005) in tumor metastasis compared to gulo KO mice on the control diet. The mean tumor weight in ascorbate supplemented mice injected with 4T1 cells was reduced by 28% compared to tumor weight in scorbutic mice. Scorbutic tumors demonstrated large dark cores, associated with increased necrotic areas and breaches to the tumor surface, apoptosis and matrix metalloproteinase-9 (MMP-9), and weak, disorganized or missing collagen I tumor capsule. In contrast, the ascorbate-supplemented group tumors had smaller fainter colored cores and confined areas of necrosis/apoptosis with no breaches from the core to the outside of the tumor and a robust collagen I tumor capsule. In both studies, ascorbate supplementation of gulo KO mice resulted in profoundly decreased serum inflammatory cytokine interleukin (IL)-6 (99% decrease, p=0.01 in the B16F0 study and 85% decrease, p=0.08 in the 4T1 study) compared to the levels in gulo KO mice deprived of ascorbate. In the B16FO study, ascorbate supplementation of gulo KO mice resulted in profoundly decreased serum VEGF (98% decrease, p=0.019 than in the scorbutic gulo KO mice). As expected, mean serum ascorbate level in ascorbate-restricted mice was 2% (p<0.001) of the mean ascorbate levels in supplemented mice. In conclusion, ascorbate supplementation hinders metastasis, tumor growth and inflammatory cytokine secretion as well as enhanced encapsulation of tumors elicited by melanoma and breast cancer cell challenge in gulo KO mice.

## Introduction

Breast cancer, the most prevalent cancer in women worldwide, and the leading cause of cancer death in women, was projected to claim the lives of approximately 39,500 women in the US in 2011 (1,2). Though treatable in early stages, once metastasis has occurred the survival rate is drastically reduced to a median of 2–3 years and treatment focuses on palliative care (3). Melanoma, another aggressive cancer, also has no viable treatment once it metastasizes from the skin to other areas of the body, such as lymph nodes, lungs, liver, brain or bone. Since 90% of cancer deaths occur secondary to metastasis, any successful anticancer treatment has to target this stage of cancer development.

Critical events in tumor cell invasion include cell attachment, proteolytic degradation of the extracellular matrix (ECM) and migration through the disrupted matrix (4). Rath and Pauling proposed that the most effective and universal approach to controlling cancer is ensuring optimal synthesis and integrity of collagen, which is dependent upon essential nutrients, such as vitamin C and lysine (5). Ascorbic acid, needed for synthesis and hydroxylation of collagen and optimal stability of the ECM, is produced by most animals but not by humans (5). Humans must obtain ascorbate by diet and cancer patients have been shown to have very low reserves of vitamin C (6,7).

Our main objective was to determine the effect of dietary ascorbate supplementation on the development of tumors in mice unable to synthesize ascorbic acid, gulonolactone oxidase (gulo) knockout (KO) mice using two models: challenging mice with breast cancer 4T1 cells into the mammary pads and challenging with injection of melanoma B16FO cancer cells intraperitoneally (IP). The 4T1 mammary tumor carcinoma model was chosen for this study as it has several characteristics that make it a suitable experimental animal model for human mammary cancer growth (8,9). The tumor cells are easily transplanted into the mammary gland so that the primary tumor grows in the anatomically correct site. Second, as in human breast cancer, 4T1 metastatic disease develops spontaneously from the primary tumor. Also the progressive spread of 4T1 metastases to the draining lymph nodes and other organs is very similar to that of human mammary cancer. The B16FO model was utilized since metastatic malignant melanoma cells, specifically B16, cells are extremely aggressive and metastasize to secondary areas of the body, such as lymph nodes, lungs, liver, brain or bone and have been successfully utilized for experimental metastasis to study the effectiveness of anticancer agents (10).

## Materials and methods

### 

#### Animals

Female Balb/C wild-type mice and mice heterozygous for the sfx mutation, a deletion in the L-gulono-γ lactone oxidase gene (gulo) were obtained from The Jackson Laboratory (Bar Harbor, ME). The gulo mice were bred to develop a homozygous gulo KO colony and were maintained on vitamin C fortified food and water. Genotyping of litters was performed through Transnetyx (Cordova, TN) and female gulo KO mice were selected from the homozygous colony for the study. Gulo KO mice selected for the study were approximately 40–42 weeks of age at the time of inoculation. In addition, a group of wild mice 14 weeks of age at the time of inoculation were used as a reference for the study. Mice were acclimated for a week before treatments, housed in standard separator cages with bedding on a 24-h light/dark schedule. All animals were cared for in accordance with institutional guidelines for the care and use of experimental animals.

#### Diet

Gulo KO mice were divided into two groups: group 1 and 2, and wild-type mice were allocated to group 3. Prior to injection with melanoma or breast cancer cells, the groups of mice were maintained for 4 weeks on the following diets. Group 1 (vitamin C deprived gulo KO mice) and group 3 (wild-type mice) were maintained on a regular diet (Laboratory Rodent Diet 5001 from Purina Mills, LLC/Test Diet) and distilled water. Group 2 (vitamin C-supplemented gulo KO mice) were provided the regular diet supplemented with 500 ppm L-ascorbyl-2-polyphosphate and distilled water with 150 mg/l ascorbic acid, 0.01 mM EDTA. The ascorbate-supplemented nutrient mix diet was milled and pressed by Purina Mills, LLC.

### Experimental design

#### Study 1

After mice had been on their respective diets for 4 weeks, 5×10^5^ murine melanoma B16FO cells in 0.2 ml PBS were injected intraperitoneally into each mouse. Mice were composed of 3 groups: group 1, ascorbate-restricted gulo KO mice (n=6); group 2, ascorbate-supplemented gulo KO mice (n=6); and group 3, wild-type mice (n=6). Mice were continued on their respective diets for another 2 weeks. The mice were then sacrificed, blood was drawn for serum analysis and peritoneal cavities were exposed and photographed. All procedures were conducted under protocols approved by the Internal Animal Care and Use Committee (IACUC).

#### Study 2

After mice had been on their respective diets for 4 weeks, 5×10^5^ 4T1 breast cancer cells in 0.2 ml PBS were injected into the mammary pad of mice. Mice were composed of 3 groups: group 1, ascorbate-restricted gulo KO mice (n=6), group 2, ascorbate-supplemented gulo KO mice (n=6) and group 3, wild-type mice (n=6). Mice were continued on their respective diets for another 2 weeks after injection. The mice were then sacrificed, blood was drawn for serum analysis and their tumors were measured, excised, weighed, photographed and processed for histology and immunohistochemistry. All procedures were conducted under protocols approved by the IACUC.

#### Metastasis grading

Mice in all groups injected IP with B16FO melanoma cells were evaluated for metastasis. They were graded based on the following scale: 0, none (only primary tumor, no secondary); 1, mild (sporadic and small metastases different from primary inoculation site); 2, moderate (obvious disseminated multiple secondary metastases); 3, severe (large, obvious disseminated multiple secondary metastases); and 4, advanced (large, obvious disseminated multiple secondary metastases with adjacent destruction and boundary loss of organs).

#### Serum analysis

Serum was processed from whole blood and stored at −80°C until analyzed. Cytokine analyses, including interleukin (IL)-6 and vascular endothelial growth factor (VEGF) were run in duplicates by Procarta^®^ Cytokine Assay Service using Procarta^®^ Cytokine kit mouse 8-plex at Affymetrix (Panomics, Santa Clara, CA). Serum ascorbate analysis was performed using the Ferric Reducing Ascorbate (FRASC) Assay kit form Biovision (Mountain View, CA).

#### Immunohistochemistry

Tumors were placed in a formalin cassette and sent to IDEXX Pathology (Sacramento, CA, USA) and HistoTox Labs Inc. (Boulder, CO, USA) for analyses. Formalin fixed samples of tumors were trimmed, processed, blocked, sectioned and stained with H&E and EVG stains, and evaluated microscopically by IDEXX Pathology. Immunohistochemistry of tumor sections was conducted by HistoTox Labs Inc. and included TUNEL stain, collagen I and IV, matrix metalloproteinase (MMP)-2 and MMP-9, Ki67, fibronectin and bcl-2.

#### Statistical analysis

The results are expressed as means ± SD, as indicated in the results, for the groups. Data were analyzed by independent sample t-test and Pearson’s correlation coefficient using MedCalc Software (Mariakerke, Belgium).

## Results

### 

#### Effect of dietary ascorbate on metastasis in mice injected with B16FO melanoma cells

**Figure 1.**
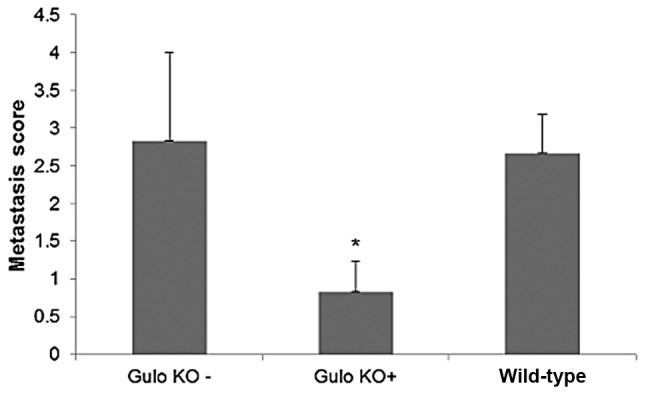
Effect of ascorbate on metastasis grade of gulo KO mice injected IP with 5×10^5^ B16FO melanoma cells (* indicates significance of p= 0.005 between gulo KO+ and gulo KO− groups). Gulo KO−, ascorbate deprived; gulo KO+, ascorbate supplemented.

**Figure 2.**
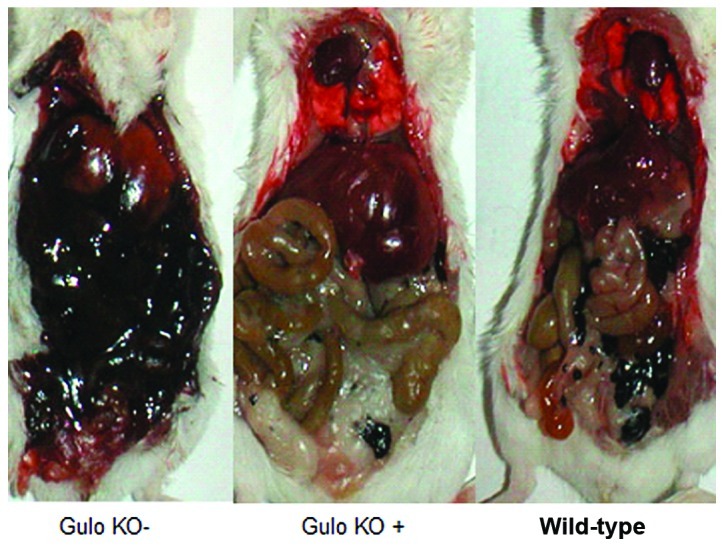
Effect of ascorbate on B16FO melanoma metastasis in gulo KO mice: gross tumor photographs of groups. Gulo KO-, ascorbate deprived; gulo KO+, ascorbate supplemented.

Ascorbate-supplemented gulo KO mice demonstrated profound and significant reduction in tumor metastasis than did the gulo KO mice on the control diet. The tumor grade was reduced by 71% (p=0.005) with ascorbate supplementation, as shown in Fig. 1. Intraperitoneal metastasis was extensive in the ascorbate depleted gulo KO mice in contrast to the ascorbate supplemented gulo KO mice and the wild-type mice, as shown in Fig. 2. A significant negative correlation (r=−0.693, p=0.026) was found between pooled metastasis grade of gulo KO mice and serum ascorbate levels.

#### Effect of dietary ascorbate on tumor growth in mice injected in the mammary pad with breast 4T1 cells

**Figure 3.**
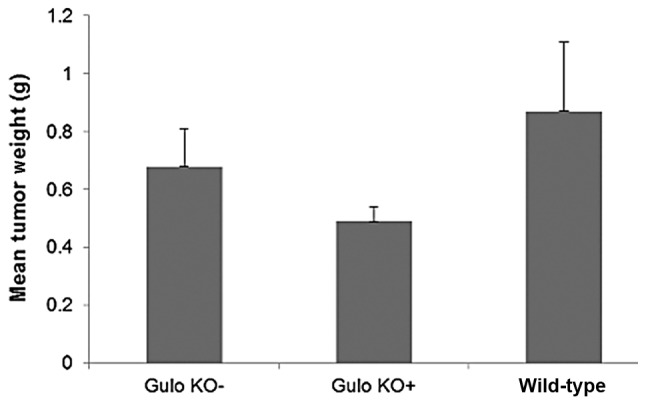
Effect of ascorbate on 4T1 breast tumor growth in gulo KO mice (SEM bars). Gulo KO-, ascorbate deprived; gulo KO+, ascorbate supplemented.

The mean tumor weight in ascorbate supplemented mice (0.49±0.13 g) was reduced by 28%, compared to tumor weight in ascorbate-deprived gulo KO mice (0.68±0.33 g), as shown in Fig. 3, but the difference did not reach statistical significance. The mean tumor weight of wild-type mice (0.87±0.35 g) was higher than that of the gulo KO mice. Mean tumor dimension for scorbutic mice (137 mm^2^) was greater than for supplemented (93.3 mm^2^) and wild-type (93.3 mm^2^) mice, but the difference did not reach statistical significance.

**Figure 4.**
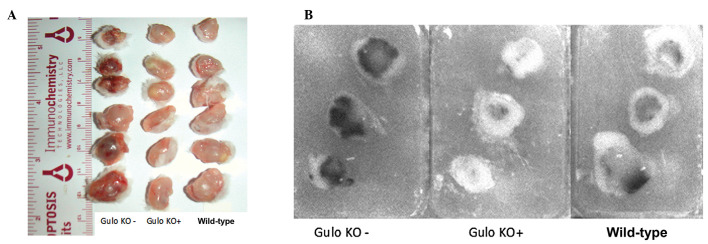
Effect of ascorbate on 4T1 breast tumor growth in gulo KO mice groups. Gulo KO−, ascorbate deprived; gulo KO+, ascorbate supplemented; 4A, gross tumor photographs of groups; 4B, paraffin sections of tumors of groups.

**Figure 5.**
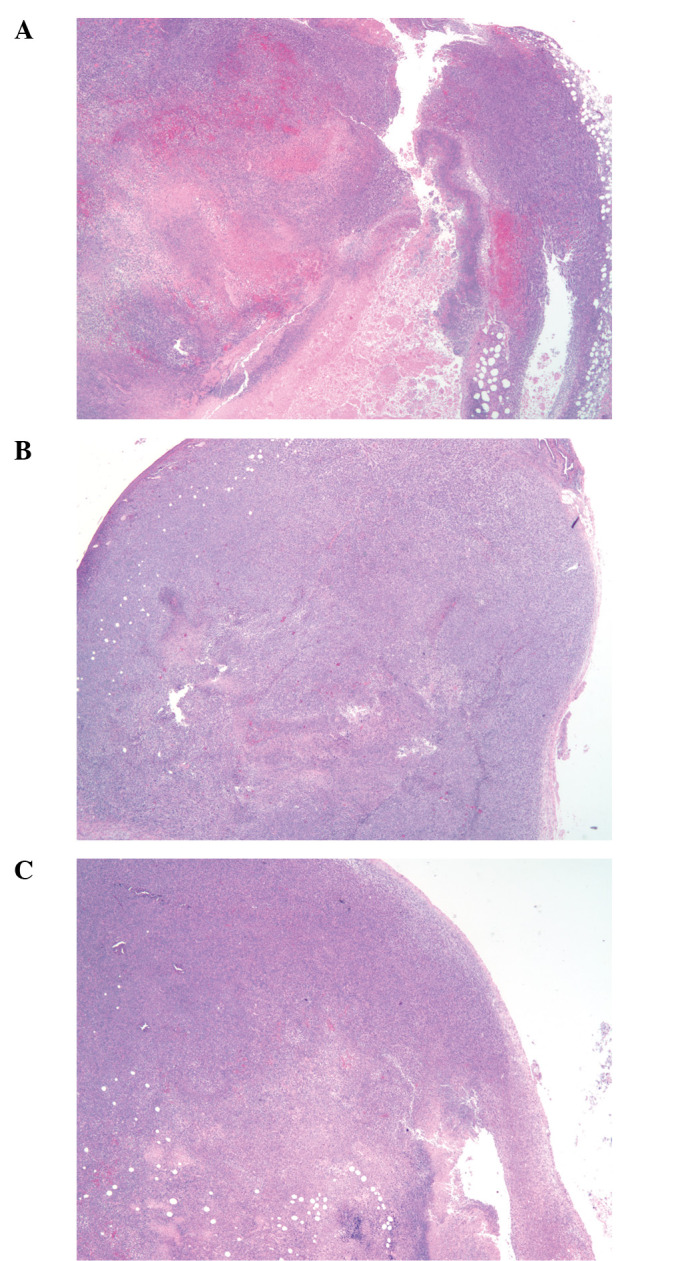
Effect of ascorbate on 4T1 breast tumor growth in gulo KO mice: H&E stains of tumors of groups. Gulo KO−, ascorbate deprived; gulo KO+, ascorbate supplemented. (A) Scorbutic gulo KO mouse; (B) ascorbate supplemented gulo KO mouse; (C) wild-type mouse; magnification, ×2.

As shown in the gross tumor photographs and paraffin sections of representative tumors from the gulo KO groups (Figs. 4 and 5), 4T1 tumors from the scorbutic groups had large dark cores in contrast to the supplemented group tumors which had smaller fainter colored cores. Tumors from wild-type mice showed an intermediate appearance. The visual difference coincided with increased core necrotic areas, which in some cases extended to and breached through the tumor surface.

### Effect of vitamin C supplementation on tumor histology: 4T1 study: Immunohistochemistry

#### Collagen I and IV

**Figure 6.**
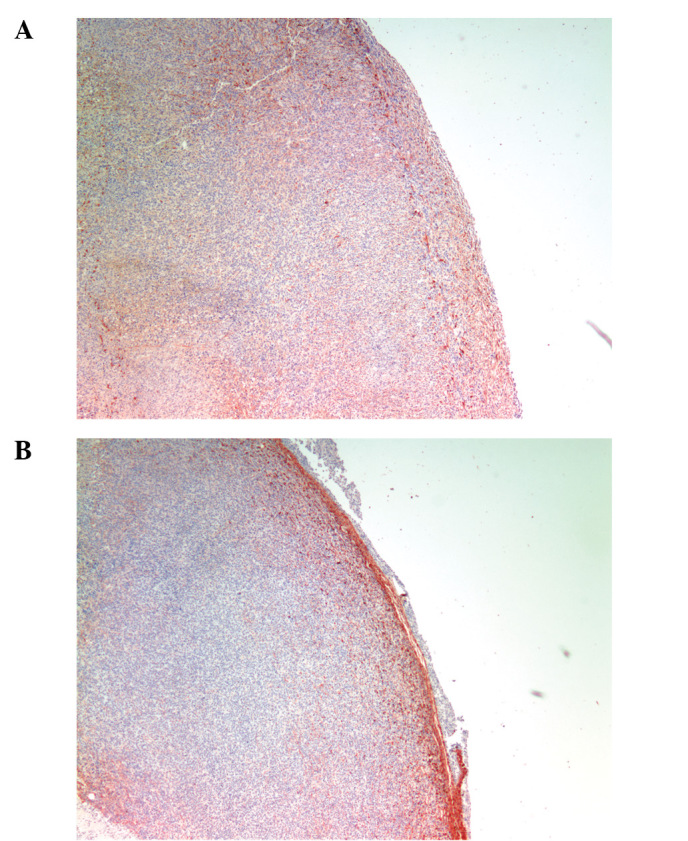
Effect of ascorbate on collagen type I in representative 4T1 tumors: IHC. (A) Scorbutic gulo KO mouse; (B) ascorbate supplemented gulo KO mouse; magnification, ×4.

**Figure 7.**
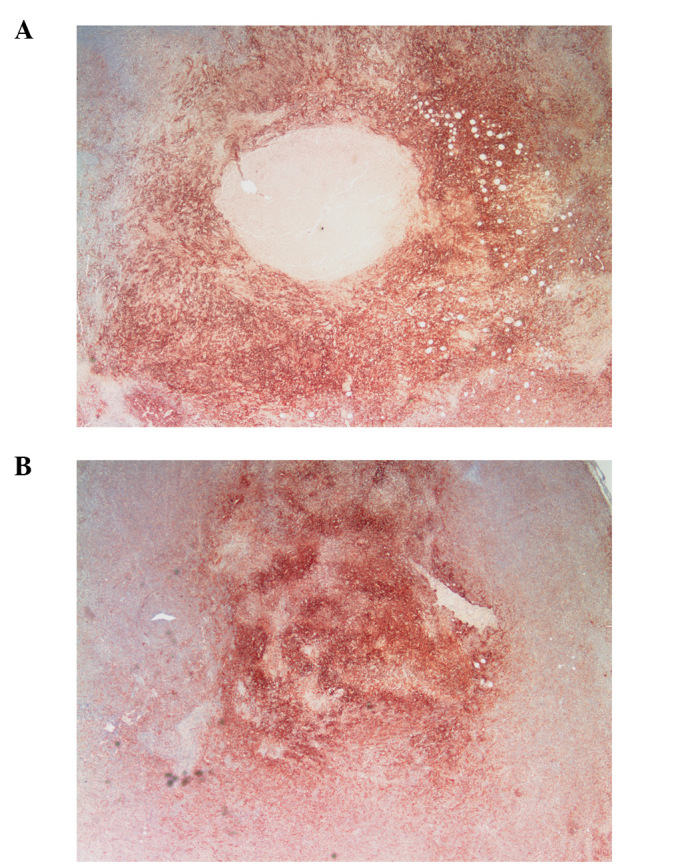
Effect of ascrobate on collagen type IV in representative 4T1 tumors: IHC. (A) Scorbutic gulo KO mouse; (B) ascorbate supplemented gulo KO mouse; magnification, 2×.

Tumors from ascorbate-supplemented mice (Figs. 6B and 7B) showed diffuse staining of collagen IV around the core and a robust collagen I tumor capsule. Scorbutic mice tumors (Figs. 6A and 7A) demonstrated a strong staining of collagen IV around the core, within the tumor, and weaker, disorganized, or missing collagen I tumor capsule. Scorbutic tumors exhibited an ‘inside-out’ collagen IV expression pattern internally, whereas vitamin C supplemented tumors were externally encapsulated with a distinct, dense collagen I capsule.

#### MMP-2 and MMP-9

**Figure 8.**
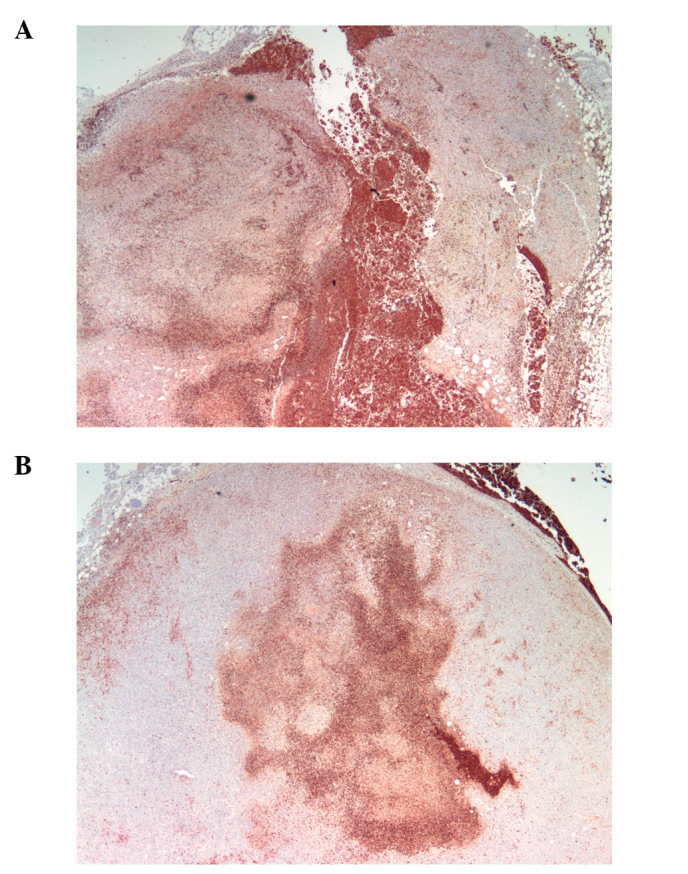
Effect of ascorbate on MMP-9 secretion in respresentative 4T1 tumors: IHC. (A) MMP-9 in scorbutic gulo KO mouse; (B) MMP-9 in ascorbate supplemented gulo KO mouse; magnification, 2×.

No significant difference in MMP-2, which was pervasive in both groups, was noted between scorbutic and ascorbate-supplemented gulo KO mice, although MMP-2 staining appeared to be less in the supplemented group (figure not shown). However, MMP-9 staining, which had a regional pattern directly overlapping necrotic areas and surrounded by apoptotic areas, differed significantly between the gulo KO groups. The scorbutic group tumors (Fig. 8A) characteristically showed an irregular pattern of necrosis in tandem with MMP-9 expression that coincided with breaches in the tumor, creating conduits and channels for viable tumor cells to escape. In contrast, the supplemented tumors (Fig. 8B) exhibited more confined areas of necrosis/apoptosis, with cancer cells in a more or less static, organized spatial organization around the core with no breaches from the core to the outside of the tumor.

#### Apoptosis: TUNEL stain

**Figure 9.**
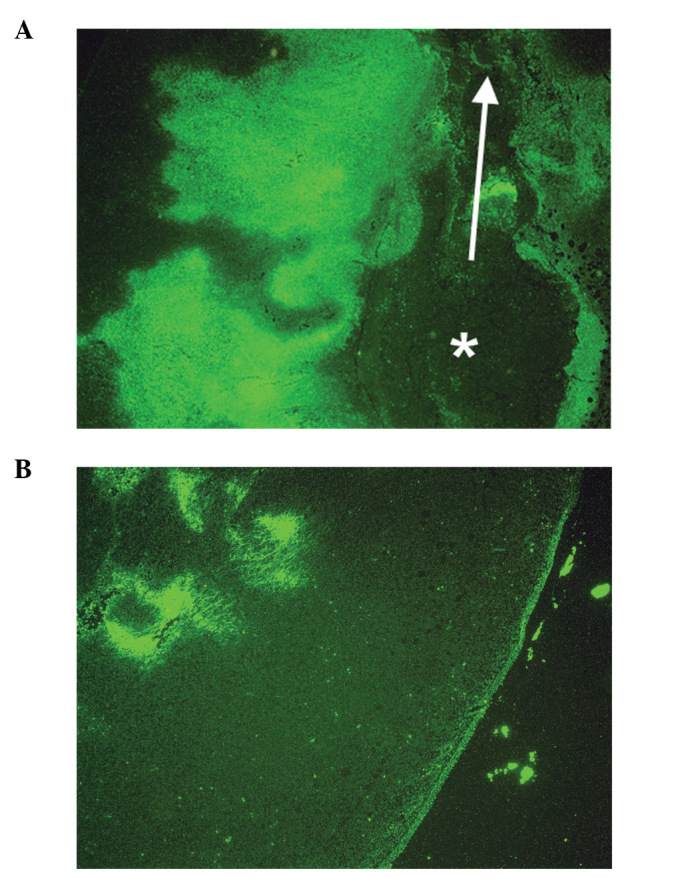
Effect of ascorbate on apoptosis in representative 4T1 tumors: IHC TUNEL. (A) Scorbutic gulo KO mouse; (B) ascorbate supplemented gulo KO mouse; magnification, 4×.

Apoptotic areas and level of apoptosis surrounded and coincided with MMP-9 area and intensity. Scorbutic group tumors (Fig. 9A) showed a pattern of apoptotic cells directly adjacent to MMP-9 staining, with necrotic areas forming patterns of breaches/conduits in the tumor from the core to the surface of the tumor. In contrast, apoptotic areas in the supplemented group tumors (Fig. 9B) were more centralized and did not breach the surface of the tumor.

#### Proliferation: Ki67

**Figure 10.**
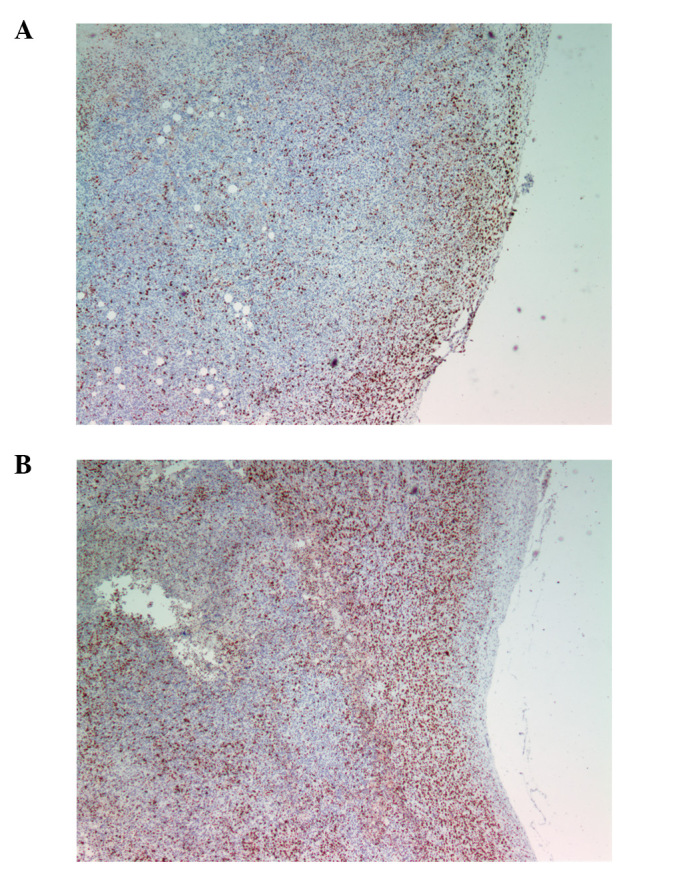
Effect of ascorbate on Ki67 expression in representative 4T1 tumors: IHC. (A) Scorbutic gulo KO mouse; (B) ascorbate supplemented gulo KO mouse; magnification, 4×.

The proliferation marker Ki67 showed similar intensity and frequency of staining between the groups except that proliferating cells were not confined by a capsule barrier in the scorbutic group and extended to the surface of the tumor (Fig. 10A). In contrast, Ki67 cells were confined and restrained by a capsule barrier in the ascorbate supplemented group (Fig. 10B).

#### Bcl-2

**Figure 11.**
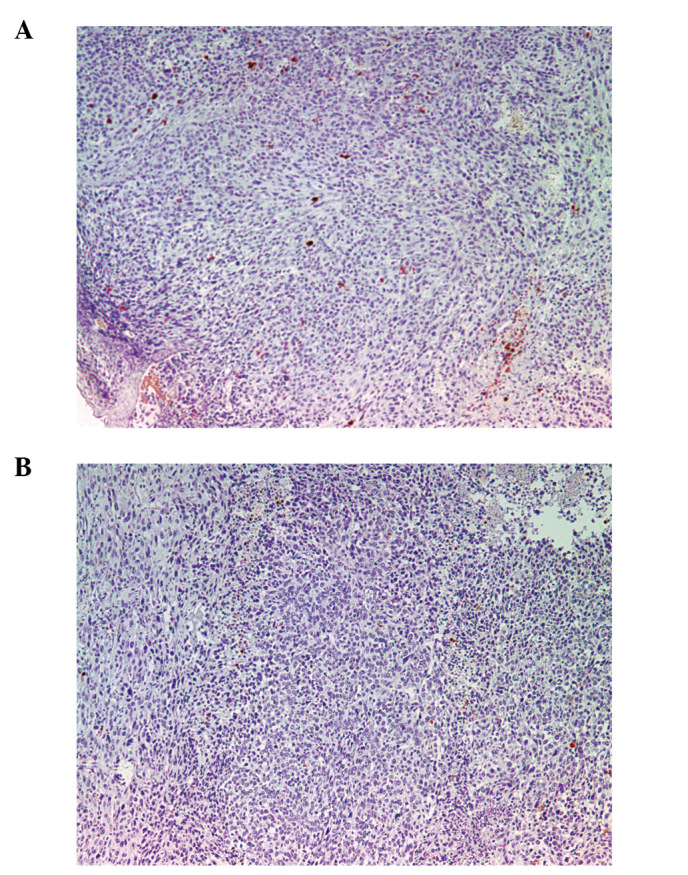
Effect of ascorbate on Bcl-2 expression in representative 4T1 tumors: IHC. (A) Scorbutic gulo KO mouse; (B) ascorbate supplemented gulo KO mouse; magnification, ×10.

More sporadic, punctate staining of Bcl-2, a pro-survival, anti-apoptotic protein, was observed in ascorbate supplemented and wild-type mice groups compared to scorbutic group tumors, but the difference was not significant. Though present, the low frequency of Bcl-2 expression would not be expected to significantly contribute to tumor regression or progression. See Fig. 11 for Bcl-2 levels in gulo KO mice groups.

#### Fibronectin

**Figure 12.**
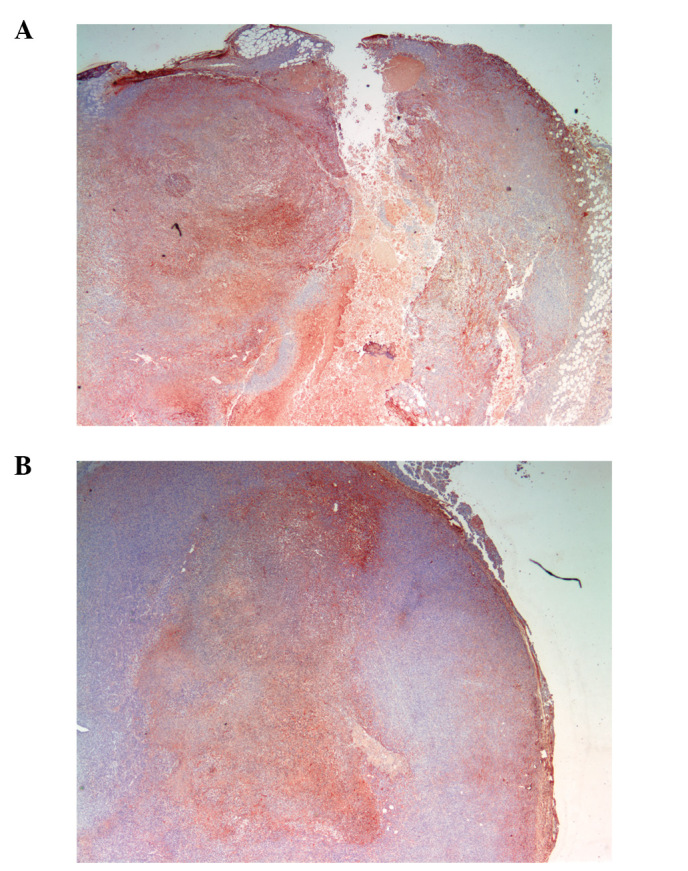
Effect of ascorbate on fibronectin expression in representative 4T1 tumors: IHC. (A) Scorbutic gulo KO mouse; (B) ascorbate supplemented gulo KO mouse; magnification, ×2.

Fibronectin staining in the scorbutic tumors was more centralized in contrast to the more peripheral staining in the ascorbate supplemented tumors, as shown in Fig. 12.

#### Effect of dietary ascorbate on weight of mice

**Figure 13.**
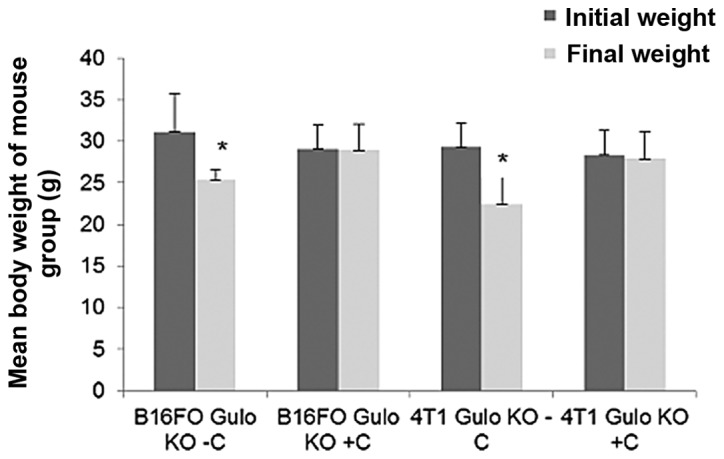
Effect of ascorbate on mean body weights of gulo KO mice in B16FO and 4T1 studies (^*^indicates significance of at least p=0.02 between gulo KO+ and gulo KO− groups).

The mean weight of mice in each group did not significantly differ at onset. However, the mean weight of ascorbate restricted gulo KO mice significantly decreased with time, showing a 19% (p=0.02) and 24% (p=0.003) decrease in weight at 6.5 weeks post injection compared to the initial mean weight prior to injection in the B16FO and 4T1 studies, respectively, as shown in Fig. 13. Ascorbate-supplemented gulo KO mice and wild-type mice maintained their weight.

#### Effect of ascorbate supplementation on serum ascorbate levels in gulo KO mice

**Figure 14.**
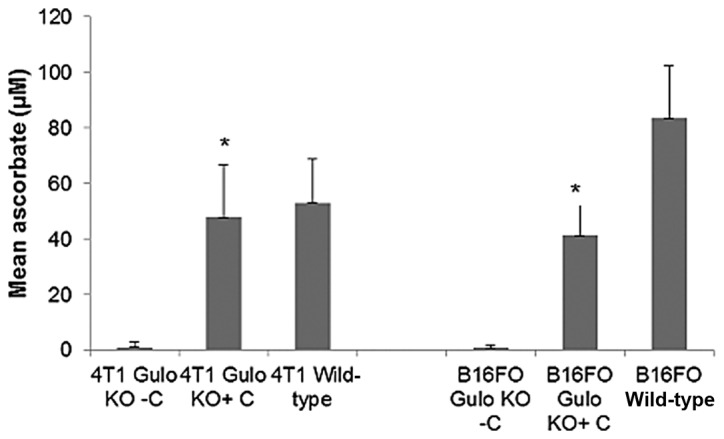
Effect of ascorbate supplementation on serum ascorbate levels in gulo KO mice in B16FO and 4T1 studies (^*^indicates significance of p<0.001 between gulo KO+ and gulo KO− groups).

As expected, ascorbate-supplemented gulo KO mice in both studies were found to have profoundly higher ascorbate levels than did the restricted gulo KO mice (Fig. 14). In the B16FO study, mean serum ascorbate level in ascorbate restricted mice (0.80±1.2 *μ*M) was 2% (p<0.001) of the mean ascorbate level in supplemented mice (41.2±11.6 *μ*M). Wild-type mice showed significantly higher ascorbate levels than either of the gulo KO mice (83.5±18.7 *μ*M). The mean ascorbate level in ascorbate supplemented mice was 49% of that in wild-type mice. In the 4T1 study, mean serum ascorbate level in ascorbate restricted mice (1.1±2.01 *μ*M) was 2% (p<0.001) of the mean ascorbate level in supplemented mice (47.9±18.9 *μ*M). Wild-type mice showed slightly higher ascorbate levels (53.1±15.7 *μ*M) than the supplemented gulo KO mice, but the difference did not reach statistical significance.

#### Effect of ascorbate supplementation on IL-6

**Figure 15.**
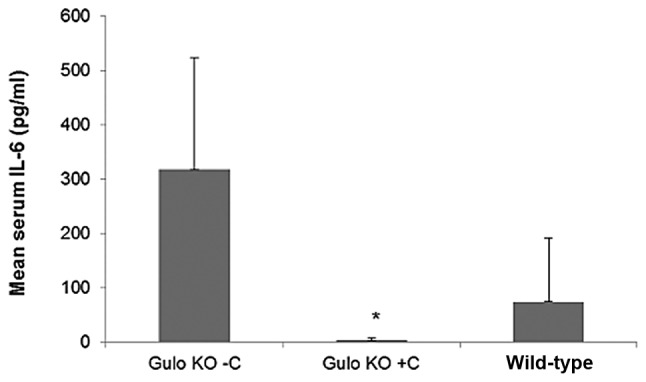
Effect of ascorbate supplementation on mean IL-6 levels in gulo KO mice in B16FO study (^*^ indicates significance of p=0.01 between gulo KO+ and gulo KO− groups).

**Figure 16.**
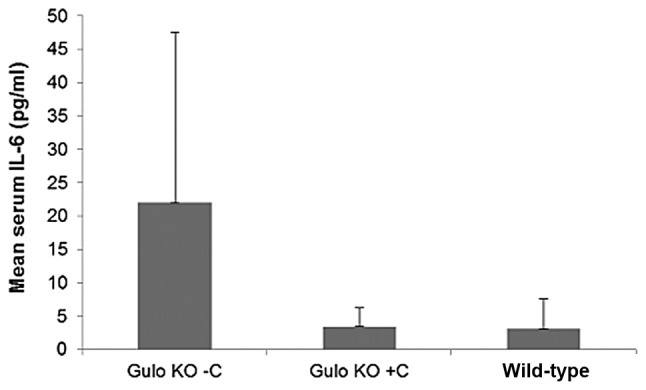
Effect of ascorbate supplementation on mean IL-6 levels in gulo KO mice in 4T1 study.

In both studies, serum inflammatory cytokine IL-6 was substantially higher in gulo KO mice deprived of ascorbate than in gulo KO mice supplemented with ascorbate. In the B16FO IP injection study, mean serum IL-6 level in the gulo KO scorbutic mouse group was 317.8±205.4 pg/ml compared to 3.4±4.75 in the ascorbate supplemented gulo KO group (99% decrease, p= 0.01), as shown in Fig. 15. In regards to the 4T1 study, ascorbate supplementation resulted in an 85% decrease from the level in the restricted gulo KO group, but the difference did not reach statistical significance, as shown in Fig. 16. Mean IL-6 levels per mouse were 3.40±2.9 and 22.0±25.64 pg/ml in the ascorbate supplemented and deprived gulo KO mice, respectively. A significant negative correlation was found between pooled ascorbate and IL-6 serum levels in mice (coefficient r=−0.7211, p=0.0024 in the B16FO study and r=−0.6062, p=0.0059 in the 4T1 study).

#### Effect of ascorbate supplementation on VEGF

**Figure 17.**
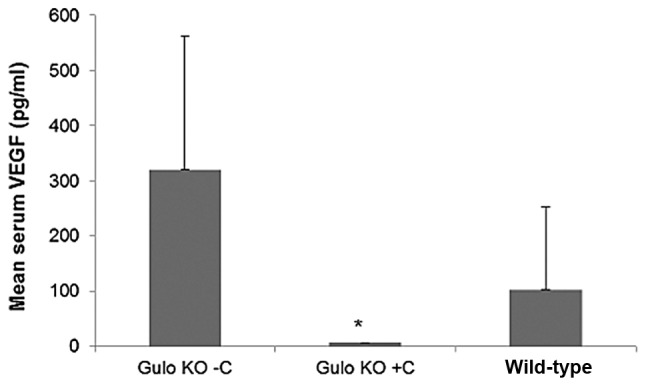
Effect of ascorbate supplementation on mean VEGF levels in gulo KO mice in B16FO study (^*^ indicates significance of p= 0.02 between gulo KO+ and gulo KO− groups).

VEGF was substantially higher in gulo KO mice deprived of ascorbate than in gulo KO mice supplemented with ascorbate. In the B16FO IP injection study, mean serum VEGF level in the gulo KO scorbutic mouse group was 321±241 pg/ml compared to 6.8±0.5 in the ascorbate supplemented gulo KO group (98% decrease, p=0.02), as shown in Fig. 17.

## Discussion

Numerous clinical studies have noted that cancer patients exhibit abnormally low plasma ascorbate levels secondary to the disease and/or treatment (6,7,11,12). A significant correlation has been demonstrated between deficient plasma ascorbate levels in cancer patients and decreased survival, as well as increased expression of inflammatory factors (11). Tumor aggressiveness has also been correlated to low ascorbate content (12). Ascorbate has been implicated in host resistance to neoplasia, at both the stromal and systemic levels.

Stromal resistance is dependent upon the host’s ability to encapsulate the neoplastic cells by forming a practically impenetrable barrier of dense fibrous tissue (5,13). Highly invasive tumors are associated with a scanty, poorly defined collagenous barrier. In contrast, collagenous barriers are more defined in tumors of moderate rapidity of growth and very abundant in slow-growing ‘contained’ atrophic scirrhous tumors (13). Degradation of adjacent matrix in the vicinity of invading neoplastic cells is dependent upon continual release of hyaluronidases, proteases and collagenases from the invading cells (4,13,14). The activity of matrix metalloproteinases (MMPs), especially MMP-9, on the degradation of the extracellular matrix plays a critical role in the formation of tumors and metastasis and high MMP-9 levels have been found to correlate with the aggressiveness of cancers, as exemplified by breast cancer and melanoma (15–17).

In our study, ascorbate supplementation of gulo KO mice injected IP with B16FO cells, demonstrated profound reduction in metastasis compared to control. B16FO metastasis was reduced by 71% in supplemented mice compared to control mice. While metastasis was not observed in the gulo KO mice that were injected with breast cancer 4T1 cells into the mammary pads, a distinct difference was observed in the size and tumor composition, especially the level of protein expression and spatial expression of the proteins between 4T1 tumors harvested from ascorbate supplemented and restricted mice. Metastasis of 4T1 cells was not expected to occur during the term of this study since tumors were harvested 2 weeks after introduction of cells while metastasis has been reported to not be detectable until the second growth phase 5–6 weeks after injection of cells (9).

In the 4T1 study, ascorbate supplementation of gulo KO mice resulted in reduction in the mean tumor weight compared to that in ascorbate-deprived gulo KO mice. Furthermore, immunohistochemical staining of tumors confirmed that this observation was not natural statistical variation inherent to *in vivo* systems, but an authentic biochemical difference in response to the presence or absence of ascorbate in the host animal. Gulo KO mice deprived of ascorbate developed large tumors with dark cores, showing more necrosis, and poorly defined borders. In contrast, gulo KO mice supplemented with ascorbate hosted smaller tumors with smaller, lighter cores, less necrosis and enhanced collagen encapsulation, signifying less metastatic potential. Apoptotic patterns from center to periphery of tumor in the scorbutic gulo KO mice, in contrast to a uniform pattern in the ascorbate supplemented gulo KO mice, suggests a more invasive and metastatic tumor structure from which cancer cells could more easily escape from the tumor mass of scorbutic mice into adjacent circulation. Furthermore, the necrotic areas that extended from the cores to breach the tumor surface in scorbutic mice were surrounded with TUNEL apoptosis staining and directly coincided with MMP-9 staining areas. This implies that irregular, asymmetric apoptosis and/or necrosis in tandem with MMP-9 expression create conduits and channels through which viable tumor cells can escape. In contrast to the irregular pattern of necrosis and breaches in the tumors found in scorbutic mice, tumors from the supplemented mice showed more confined areas of necrosis and apoptosis around the core with no breaches from the core to the outside of the tumor.

Bonfil *et al*(18) in studying the role of intratumoral necrosis in cell detachment and metastasis using non-metastatic murine mammary adenocarcinma M3 and its metastatic variant MM3 reported similar patterns to what we observed in mammary 4T1 tumors from ascorbate supplemented versus scorbutic gulo KO mice. The histological studies of Bonfil *et al* revealed a central necrosis limited by an uninterrupted peripheral ring of well-preserved cells in M3, in contrast to alternated necrotic and non-necrotic areas in MM3. They concluded that the distribution of necrosis within the primary tumor was responsible in part for the development of metastases (18). Furthermore, in a subsequent study, they noted that tumor necrosis was an important source of gelatinase/type IV collagenase, mainly in its 92 kDa form, and thus played a major role in tumor invasion (19).

In addition to promoting progression of cancer, elevated pro-inflammatory cytokine levels have been associated with a variety of pathologies, such as fatigue, depression and cachexia (20–23). Levels of serum cytokines, such as IL-6, IL-1β, IL-1α, IL-8, IL-12p40, IL-13, GM-CSF, monocyte chemoattractant protein (MCP)-1, macrophage inflammatory protein (MP)-1α, MP-1β, IFNα, tumor necrosis factor (TNF)-α, epidermal growth factor, VEGF and TNF receptor II are reported to be significantly higher in patients with breast cancer and with resected high-risk melanoma than in healthy controls (24,25). These factors promote cancer growth and stimulate angiogenesis, which lead to cancer growth and metastasis in these and other cancers (26,27). Clinical studies have shown elevated IL-6 levels in breast cancer to be associated with poor breast cancer prognosis (28–30) and to increase with tumor grade and number of metastatic sites (29). Similarly, significantly increased levels of serum IL-6 in malignant metastatic melanoma patients have been found to be correlated to the tumor burden in these patients (31–33). Ascorbate supplemented gulo KO mice had profoundly reduced levels of IL-6 compared to ascorbate-deprived gulo KO mice in both the melanoma B16FO and breast cancer 4T1 studies. Furthermore, negative correlations were found between IL-6 secretion and ascorbate levels in both studies from pooled data. The pro-angiogenic factor VEGF, which is critical for both primary tumor growth and metastasis, was also found to be significantly higher in scorbutic mice challenged with melanoma B16FO cells than in ascorbate-supplemented mice, which was reflected in the higher metastasis grade of the scorbutic group. Of interest, weight loss occurred in the gulo KO mice deprived of ascorbate but not in those supplemented with ascorbate. The weight loss observed in cancer patients, secondary to the inflammation-associated cachexia, mirrors the weight loss observed in the scorbutic mice with high IL-6 levels.

The comparative ascorbate serum levels in the gulo KO groups in both studies support the claim that the beneficial effects observed in the ascorbate-supplemented mice were due to significantly increased serum ascorbate levels. Oral supplementation of vitamin C to levels included in diets for mammals that do not generate endogenous ascorbate, such as guinea pigs, and in the drinking water did not achieve pharmacological levels of ascorbate in the gulo KO mouse serum. Measured levels of serum ascorbate from supplemented mice were within the normal physiological range reported throughout the literature (34). Despite being 10-fold lower than pharmacological or therapeutic concentrations, this normal physiological level of serum ascorbate sustains a biochemical environment less conducive to metastasis and growth of melanoma and breast cancer, respectively. In contrast, the resultant reduced host resistance and less effective response of scorbutic mice encouraged metastasis and tumor growth to a degree that made normal supplementation appear therapeutic in its activity. In both the melanoma and breast cancer studies, ascorbate-depleted gulo KO mice were found to have profoundly lower (2%) ascorbate levels than found in ascorbate supplemented mice. Furthermore, a significant negative correlation was found between pooled metastasis grade of gulo KO mice and serum ascorbate levels in the B16FO study. A previous mechanistic study showed that ascorbic acid reduced leukemia HTLV-1 cell proliferation and induced apoptosis by the modulation of p53, p21, Bcl-2 and Bax (35).

Mean ascorbate level was slightly higher in wild mice compared to supplemented gulo KO mice in the 4T1 study and significantly higher in the B16FO study. The higher ascorbate levels in wild-type mice are probably due to the age difference between gulo KO and wild-type mice. In addition, younger (3 months old) Balb/c mice have been reported to grow significantly larger tumors than older (9+ months old) mice (36). These findings would probably explain the higher metastasis grade and larger tumor weight observed with the wild-type mice compared to the supplemented gulo KO mice. Though this was not the focus of the study, the use of wild-type reference mice that were considerably younger (by 6 months) than the experimental set was a limitation. In a previous study of B16FO growth/metastasis using wild-type and gulo KO groups of mice both approximately 36–40 weeks of age, no statistical difference was observed between tumor growth in wild-type and supplemented gulo KO mice (37).

In conclusion, ascorbate supplementation resulted in decreased metastasis elicited by melanoma IP challenge in gulo KO mice and reduced tumor growth and enhanced encapsulation of tumors resulting from breast cancer challenge. Tumor encapsulation is critical in curbing invasion and metastasis of malignant cells. In addition, ascorbate supplementation modulated inflammatory cytokine secretion. Scurvy, which results from severe dietary lack of ascorbate, exhibits generalized stromal changes identical to local stromal changes observed in cancer in the immediate vicinity of invading neoplastic cells (38). Thus, ascorbate supplementation to cancer patients has been proposed to reverse their scorbutic symptoms and treat the cancer (13,39). The results of this study support this proposal.
